# Understanding foot-and-mouth disease virus transmission biology: identification of the indicators of infectiousness

**DOI:** 10.1186/1297-9716-44-46

**Published:** 2013-07-03

**Authors:** Margo E Chase-Topping, Ian Handel, Bartlomiej M Bankowski, Nicholas D Juleff, Debi Gibson, Sarah J Cox, Miriam A Windsor, Elizabeth Reid, Claudia Doel, Richard Howey, Paul V Barnett, Mark EJ Woolhouse, Bryan Charleston

**Affiliations:** 1 Centre for Immunity, Infection and Evolution, University of Edinburgh, Edinburgh EH9 3JT, United Kingdom; 2 Royal (Dick) School of Veterinary Studies, University of Edinburgh, Easter Bush, Midlothian EH25 9RG, United Kingdom; 3 The Pirbright Institute, Surrey GU24 0NF, United Kingdom; 4 Institute of Genetic Medicine, Newcastle University, Newcastle upon Tyne NE1 3BZ, United Kingdom

## Abstract

The control of foot-and-mouth disease virus (FMDV) outbreaks in non-endemic countries relies on the rapid detection and removal of infected animals. In this paper we use the observed relationship between the onset of clinical signs and direct contact transmission of FMDV to identify predictors for the onset of clinical signs and identify possible approaches to preclinical screening in the field. Threshold levels for various virological and immunological variables were determined using Receiver Operating Characteristic (ROC) curve analysis and then tested using generalized linear mixed models to determine their ability to predict the onset of clinical signs. In addition, concordance statistics between qualitative real time PCR test results and virus isolation results were evaluated. For the majority of animals (71%), the onset of clinical signs occurred 3–4 days post infection. The onset of clinical signs was associated with high levels of virus in the blood, oropharyngeal fluid and nasal fluid. Virus is first detectable in the oropharyngeal fluid, but detection of virus in the blood and nasal fluid may also be good candidates for preclinical indicators. Detection of virus in the air was also significantly associated with transmission. This study is the first to identify statistically significant indicators of infectiousness for FMDV at defined time periods during disease progression in a natural host species. Identifying factors associated with infectiousness will advance our understanding of transmission mechanisms and refine intra-herd and inter-herd disease transmission models.

## Introduction

Foot-and-mouth disease virus (FMDV), a member of the *Aphtho*v*irus* genus within the Picornaviridae family, is the causative agent of foot-and-mouth disease (FMD), one of the world’s most important infectious animal diseases, responsible for huge global losses of livestock production and trade, as well as frequent and highly disruptive large-scale epidemics [1,2]. The disease is characterised by a short lasting fever, epithelial lesions on the tongue, dental pad and inner mouth area leading to excessive salivation and drooling and lesions on the feet causing lameness. Secondary infection of epithelial lesions can significantly increase the severity of disease [3,4].

There are seven immunologically distinct serotypes and more than 60 antigenic variations [5,6] and many are endemic in large parts of Asia, Africa and South America [7]. Here, we focus on serotype O, which is the most prevalent serotype globally and shown to be transmitted by several different routes. One of the most common routes of transmission in ruminants is by direct contact between infected and naïve animals. Indirect contact also occurs by mechanical transfer via people, wild animals and birds, vehicles, fomites and animal products e.g. milk or meat products [8-13]. The virus may also spread by inhalation of infectious droplets and droplet nuclei originating mainly from the breath of infected animals [14] which can be wind borne [15]. Wind borne transmission occurs infrequently, as it requires particular climatic and epidemiological conditions [16-18].

A recent publication [19] reported the results of experimental studies of direct FMDV transmission in cattle. The results of that study suggested that conditions promoting transmission exist for only a brief period and showed that infectiousness is a complex phenomenon related not just to virus dynamics but also to host responses and clinical signs, which is consistent with a common but rarely tested expectation that disease signs may be functionally linked to infectiousness. Prior to this research, studies into FMDV transmission had used proxy measures for infectiousness (for example the detection of virus in the blood or other tissues) rather than directly demonstrating transmission to another animal. Recent results [19] highlighted that cattle infected with FMDV are substantially less likely to be infectious before showing clinical signs than was previously realized. As such there is a need for more robust empirical evidence on relationships between clinical signs and infectiousness.

The aim of the present study was to utilize the relationship between the onset of clinical signs and direct contact transmission of FMDV to identify possible predictors of the onset of clinical signs as well as identify candidates for preclinical screening in the field. Such information will advance our knowledge of the transmission mechanisms and improve the model predictions that are used in disease control. The assumption that the likelihood of transmission is decreased if control can be implemented just 24 h earlier provides strong support for investment in the development of practical tools for preclinical diagnosis. If we can identify infected cattle before they show signs of disease using tests in the laboratory then perhaps these can be used in the field during an outbreak. Measures of concordance between qualitative real time (qRT)-PCR results and virus isolation results were also determined in each experiment. These measures of concordance are useful in evaluating the performance of both methods of virus detection.

## Materials and methods

Details of the methods used in this paper have been published elsewhere [19] but are described in brief below. All experiments were approved by the Institute’s ethical review process and were in accordance with national guidelines on animal use.

### Animal experiments and samples

Four individual animal experiments using 100–150 kg Holstein Friesian calves were performed. For each experiment, two animals (referred to later as inoculates) were selected at random, and were needle challenged intradermolingually (1) with 1 × 10^5.7^ TCID_50_ of cattle adapted FMDV O UKG 34/2001. Forty eight hours after challenge naïve animals (“donors”, 2 animals for each of 4 experiments) were introduced to the inoculates and were challenged by direct contact exposure for 24 h. The inoculates were removed from the study and the animals exposed to infection (*n* = 8) were used to attempt transmission to further naive cattle (“recipients”) at two, four, six and eight (in experiments 3 and 4 only) days post infection for a period of 8 h each time. A total of 28 recipients were used in this study design.

Individual donors were examined daily for the presence of clinical signs (lesions in the mouth, tongue, snout, feet and the presence of nasal discharge). Rectal temperature was also recorded. Blood and nasal fluid samples were taken daily for the first 8 days following challenge and then every other day for up to 14 days after challenge. The samples were transferred immediately to the laboratory for processing; nasal fluid was stored at −80°C and heparinised blood aliquoted and stored for subsequent virus detection in bovine thyroid cells (BTY) culture as described earlier [19]. Aliquots of serum were stored at −80°C for subsequent total antibody (Ab) detection, nucleic acid extraction and analysis by qRT-PCR. Samples of oropharyngeal fluid (OPF) were collected by probang cup from all the animals before challenge and thereafter from the donors daily for the first week and fourteen days after challenge. All probang samples were stored at −80°C for subsequent virus detection using BTY cell culture and genome detection using real-time quantitative PCR.

Several air samples using multiple devices were collected simultaneously, each hour, during all but 2 of the 28 challenge periods. Air samples were collected using an all-glass Cyclone sampler (operated for 5 min at a flow rate of approximately 390l/min) and an all glass Porton impinger sampler (operated for 5 min at a flow rate of 11l/min). These sampling periods were determined as the optimal sampling configurations for the instruments [20-22]. The collecting media employed in these samplers was modified eagle’s medium (MEM) -HEPES with antibiotics and 0.1% (w/v) BSA [16,23]. The concentration of virus per litre of air was determined by endpoint titration for each particular air-sample, which was multiplied by the volume of the collecting fluid and the flow of the sampler. The amount of infectivity recovered was expressed as the total amount (50% tissue culture infectious dose, TCID_50_) of airborne FMDV per animal per challenge period (8 hrs).

### Virus detection

Live virus was detected in the biological samples collected (heparinised blood, nasal swabs, OPF) and in the collecting media from the air samples using BTY primary cell cultures [16,23,24]. Given the large number of samples taken, they were first screened to determine the presence of virus, and then a tenfold dilution series of those verified to be virus positive were made and each dilution inoculated onto five BTY tubes. Titres were calculated by the Karber equation according to Lennette [25]. The specificity of any cytopathic effect was confirmed by an antigen capture ELISA [26-28].

### Viral nucleic acid purification

RNA (200 μL of sample, mixed with 300 μL of MagNA Pure LC total nucleic acid Lysis/Binding Buffer) was extracted using the MagNA Pure LC total nucleic acid isolation kit (Roche, UK) and an automated nucleic acid robotic workstation according to the manufacturer’s instruction (MagNA Pure LC, Roche, UK). The samples were eluted in a volume of 50 μL and stored at −80°C for later analysis.

### FMDV RNA standards and reverse transcription

As these experiments were performed with the O UKG 34/01 isolate, a homologous RNA standard was used (synthesized in vitro from a plasmid containing a 500 base pair insert of the internal ribosomal entry site (IRES) of FMDV O UKG 34/01) as described by Quan et al. [29]. The reverse transcription was performed as previously described [29-31].

### Quantitative RT-PCR

To determine the amount of FMDV RNA in extracts of the total nucleic acid from blood, OPF, and nasal fluid a qRT-PCR was performed according to the methodology previously described [30,32]. In the PCR reaction, primers SA-UK-IRES-248F (50-AAC CAC TGG TGA CAG GCT AAG G-30)/SA-UK-IRES-308R (50-CCG AGT GTC GCG TGT AC CT-30) and a UK-IRES- 271T (6-FAM-TGC CCT TTA GGT ACC C-MGB) TaqMan® Minor Groove Binding probe (Applied Biosystems) were used, as this primer/probe set was designed for optimum detection of the FMDV O UKG 2001 virus [29]. The PCR was performed on a Stratagene® MX3005P™ QPCR system using MXPro-MX3005 v 3.00 Build 311 software (Stratagene, UK), and fifty PCR cycles were carried out. Once obtained, results and amplification plots were analysed and standard curves constructed from cycle threshold values [32-34] of the RNA standard dilutions, to provide a measure of the number of FMDV genome copies.

### Assay for FMDV specific antibodies

Serum samples were tested for the presence of antibodies to FMDV using a liquid phase blocking ELISA (LPBE) [35,36].

### Assay for interferon detection

Type-I interferon (IFN) biological activity was measured in serum samples from donor animals by using an Mx/chloramphenicol acetyltransferase (Mx/CAT) reporter gene assay [37].

### Database management and statistical analysis

Table 1 contains a list of all of the nonclinical variables measured during the course of this experiment. Examination of the structure of the data required that some variables be categorised for the purpose of statistical analysis. Cut-off levels for some variables (blood VI, blood qRT-PCR, OPF VI, OPF qRT-PCR) were determined using Receiver Operating Characteristic (ROC) curve analysis [38]. A threshold of VI > 4 log_10_ TCID_50_ per mL of nasal fluid was set because values below this threshold may be misleading and may represent low viral titres or insufficient sample collection due to limited nasal secretions. The baseline for Type 1 IFN of >1 IU per ml serum had been established previously [39]. For all variables additional “appearance” “onset” and lag variables were generated. “Appearance” identifies the first appearance of a given variable on a given day. “Onset” identifies the first appearance of the variable on a given day above the pre-determined cut-off. Lag variables were generated to identify the “appearance” or “onset” of a given variable either 1 day or 2 days prior. “Onset of any clinical signs” was defined as the appearance of any of nasal discharge, lameness, or lesions on the feet, mouth, tongue. For the purposes of this analysis temperature was analysed separately to the other clinical signs.

**Table 1:** List of virological, immunological and environmental variables generated in this study and included in statistical analysis

**Variable**	**Description**
OPF VI	Quantity of live virus in oropharyngeal fluid (OPF) (log_10_ TCID_50_/mL)
OPF qRT-PCR	Quantity of FMDV genome copies in OPF (log_10_ copies/mL)
Blood VI	Quantity of live virus in the blood (log_10_ TCID_50_/mL)
Blood qRT-PCR	Quantity of FMDV genome copies in blood (log_10_ copies/mL)
IFN	Type-1 interferon in serum (IU/mL)
Nasal fluid VI	Quantity of live virus in the nasal fluid (log_10_ TCID_50_/mL)
Antibodies	FMDV-specific antibodies detected in serum (titre/mL)
Air sample	Total airborne FMDV per animal per challenge period

**Figure 1:**
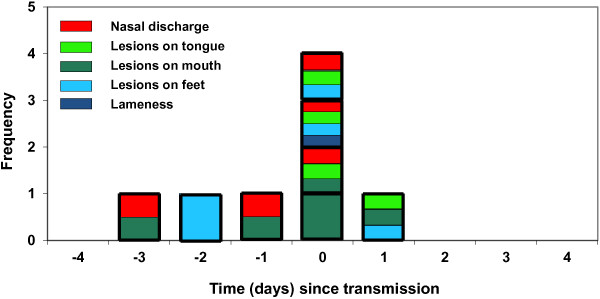
**Onset of clinical signs.** The time (in days) since transmission when clinical signs appear for donors with successful transmission events (*n* = 8 transmission events for *n* = 7 donors). The bars for each donor (bold lines) are colour coded with the first clinical sign(s) reported for each donor.

Identification of the predictors of the onset of clinical signs was performed using a Generalised Linear Mixed Model (GLMM). The onset of clinical signs (as opposed to transmission) was chosen as the response variable as it was previously shown that there is a significant association between transmission and the onset of clinical signs [19] (see Figure 1). Onset of clinical signs refers to whether or not the donor cow developed clinical signs on the given day. These clinical observations were confirmed later by detection of live virus from the recipient. No subclinical infections were observed.

Only data up to and including day 8 were used in the statistical analyses as after day 8 data was not collected daily. In addition, all donor animals had exhibited clinical signs by day 8. To account for the repeated measures structure in the data (i.e. daily sampling) analyses using multiple explanatory variables were performed using a GLMM with covariance pattern model (Proc Glimmix SAS version 9.3, SAS Institute Inc., Cary, NC). The GLMM was fitted with a binomial distribution and a logit link function. The response variable was the presence or absence of any clinical signs. Donor was fitted as the sole random effect, other variables were fitted as fixed effects. Given the small sample size (n=8 donors) only single factor models were generated. In total 63 variables were analysed.

Diagnostics were performed and plots of residuals were examined, confirming the goodness-of-fit of each model. Odds ratios (OR) and their associated 95% confidence intervals were estimated in the final models for factors statistically significantly associated with the onset of clinical signs. The generalised chi-square/DF (*χ*^2^/DF) was used to compare the fit of different models. Unfortunately no significance test of model fit is available, however, the *χ*^2^/DF can identify models that given their fixed and random effect specifications more (or less) closely meet the specified distribution (binomial logit). The closer the *χ*^2^/DF is to unity the better the model and data meet the assumed residual distribution.

Virus in the blood and OPF was measured using both the virus isolation method (from heparinised blood samples) and the qRT-PCR method (from serum samples). The relationship between the two methods was examined by looking at the temporal trends using PROC LOESS (SAS version 9.3). LOESS is a nonparametric method for estimating local regression surfaces. In addition, agreement between the two methods was examined using Cohen’s kappa (StatXact v.8, Cytel Software Corp, Cambridge, MA, USA).

Preclinical predictors were identified in the data by examining the virological and immunological variables in the time frame surrounding the onset of clinical signs. Data from ±4 days from the onset of clinical signs for each donor animal were included.

Prior to analysis, it was specified that results with *p* <0.05 would be reported as exhibiting formal statistical significance.

## Results

### Associations with transmission

As previously described [19] there were 28 attempts to transmit the disease from donor to recipient animals over the 8h periods, 8 (29%) of which were successful in transmitting disease. Six of these transmissions occurred on either day 4 or day 6 since exposure of the donor to the virus. Only one transmission event occurred on each of day 2 and day 8. One donor transmitted the disease on two occasions, days 4 and 6 post exposure to the virus. One cow failed to transmit FMDV, even though FMDV was detected in the nasal fluid (NF) and oesophageal-pharyngeal fluid (OPF), although not the blood.

Transmission was significantly associated (Fisher’s exact = 6.16; *p* = 0.021) with the onset of clinical signs (Figure 1). The peak at time 0, illustrates that for most cows transmission occurs on the same day as clinical signs appear. Only one transmission event occurred prior to the onset of clinical signs. However, this animal showed overt signs the next morning, approximately 16 h after the end of the successful challenge period. The clinical signs observed were varied: nasal discharge and lesions in the mouth or tongue were the most frequently reported “first” signs (Figure 1).

Associations between air sample and transmission could only be tested on days in which transmission was attempted as air samples were not recorded daily (Table 2). Data were recorded as the total amount (TCID_50_) of airborne FMDV per animal per challenge period (8 h). Data were available for only 26 transmission days, 2 days were missing. The total amount of virus in the air ranged from 3–4.5 TCID_50_, therefore values for airborne virus were treated as either detected or not detected. A positive air sample was statistically significantly associated with transmission of FMDV (Table 2).

**Table 2:** GLMM air sampling

	**Transmission**		**Estimated effect**	**se**	**p**	**odds**	**95% CI**
Air sample	no	yes	0.948	0.821	0.021	19.9	1.65-240
positive	2	5					
negative	17	2					

Odds ratios and 95% CI for the association between a FMDV virus detected in the air and the transmission of FMDV.

### Cut-off levels

**Figure 2:**
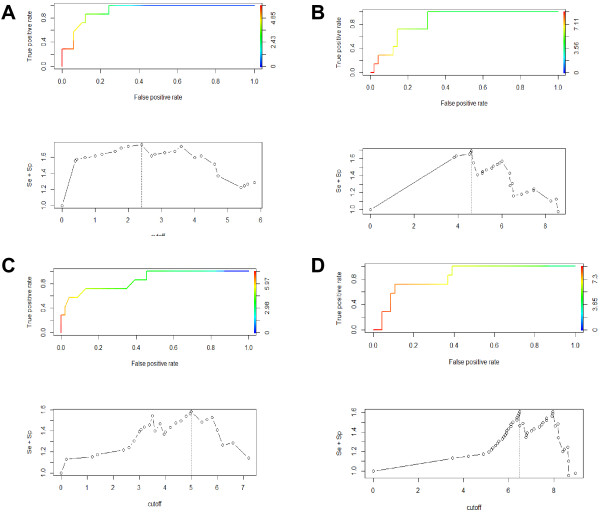
**Threshold identification by Receiver Operator Curves (ROC) analysis.** Receiver Operator Curves (top graph) and estimated cut-off (bottom graph) for **(A)** Blood VI, **(B)** Blood qRT-PCR, **(C)** OPF VI and **(D)** OPF qRT-PCR. The cut-off is defined as the value that maximizes the sensitivity + specificity.

Receiver Operating Characteristic (ROC) curve analysis was used to determine the best cut-off for the virological variables measured in this study (Figure 2). The cut-off chosen was one that maximized the sum of the sensitivity and specificity. The cut-off value for each variable is shown in Figure 2. The cut-off values for blood VI, blood qRT-PCR, OPF VI and OPF qRT-OCR were 2.4 log_10_ TCID/mL, 4.6 log_10_ copies/mL, 5.0 log_10_ TCID_50_/mL and 6.5 log_10_ copies/ml respectively.

### Predictors of transmission and the onset of clinical signs

Table 3 contains the final GLMM models for the onset of clinical signs. Of the 63 factors tested, 18 were significant at p < 0.05. All the factors identified were significant risk factors (odds ratio>1). Only single factor models are presented as the limited data restricted the power to create multivariate models. High levels of virus in OPF and nasal fluid are significant risk factors for the onset of clinical signs, particularly the onset of levels above the cut-off for both OPF and nasal fluid. High levels of virus in the blood were also significant risk factors for the onset of clinical signs (Table 3). However, unlike OPF and nasal fluid there may be a lag between levels of virus in the blood and the onset of clinical signs. Significant associations were also found with Type 1 IFN levels >1 where the onset of clinical signs was associated with Type 1 IFN levels >1 (Table 3).

**Table 3:** GLMM Onset of clinical signs

**Variable**	**Estimated effect**	**se**	**p**	**odds**	**95% CI**	***χ*2/DF**
OPF VI						
>5.0 log_10_ TCID_50_/mL	1.918	0.791	0.020	6.81	1.38-33.6	1.01
<5.0 log_10_ TCID_50_/mL	-	-				
Onset OPF VI > CT						
yes	2.410	0.974	0.016	11.1	1.59-77.9	1.02
no	-	-				
OPF qRT-PCR						
>6.5 log_10_ copies/mL	2.670	1.021	0.013	14.4	1.83-114	0.99
<6.5 log_10_ copies/mL	-	-				
Onset OPF qRT-PCR > CT						
yes	2.254	0.916	0.017	9.53	1.53-59.4	1.02
no	-	-				
Blood VI						
>2.4 log_10_ TCID_50_/mL	2.681	0.867	0.004	14.6	2.53-84.4	1.11
<2.4 log_10_ TCID_50_/mL	-	-				
Appear -1D blood VI > 0						
yes	3.325	0.994	0.002	27.8	3.83-202	1.08
no	-	-				
Appear -2D blood VI > 0						
yes	1.990	0.938	0.038	7.31	1.12-47.6	1.08
no	-	-				
Onset blood VI > CT						
yes	2.503	0.953	0.011	12.2	1.82-82.0	1.15
no	-	-				
Onset -1D blood VI > CT						
yes	4.502	1.173	0.003	90.2	8.69-936	1.11
no	-	-				
Blood qRT-PCR						
>4.6 log_10_ copies/mL	3.122	1.063	0.005	22.7	2.67-193	1.02
<4.6 log_10_ copies/mL	-	-				
Appear -1D blood qRT-PCR > 0						
yes	3.325	0.994	0.001	27.8	3.83-202	1.08
no	-	-				
Onset blood qRT-PCR > CT						
yes	2.600	0.925	0.006	13.5	2.13-85.2	1.06
no	-	-				
Onset -1D blood qRT PCR > CT						
yes	3.212	1.038	0.003	24.8	3.12-197	1.12
no	-	-				
Nasal fluid VI						
>4 log_10_ TCID_50_/mL	1.829	0.777	0.023	6.23	1.31-29.7	1.03
<4 log_10_ TCID_50_/mL	-	-				
Appear of nasal fluid VI > 0						
yes	2.917	0.927	0.002	18.5	2.91-118	1.06
no	-	-				
Onset nasal fluid VI > CT						
yes	2.511	0.972	0.012	12.3	1.77-85.6	1.02
no	-	-				
Type 1 IFN						
>1IU/mL	1.575	0.762	0.044	4.83	1.04-22.4	1.03
<1IU/mL	-	-				
Onset Type 1 IFN > CT						
yes	4.06	1.092	0.0004	57.9	6.56-52.0	1.06
no	-	-				

Estimated effects, odds ratios and 95% CI for significant (p<0.05) factors in the final generalised linear mixed models (GLMM). CT, estimated cut-off from ROC analysis. -1D/-2D represents appearance or onset of a given factor 1 or 2 days (D) prior.

### VI vs qRT-PCR measurements

**Figure 3:**
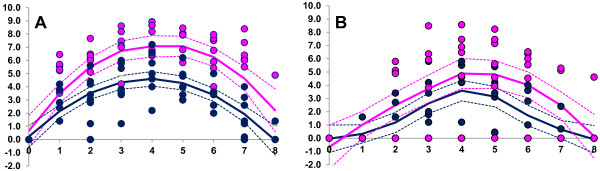
**Virus isolation (VI) vs qRT-PCR.** Nonparametric regression comparing the two methods of virus detection (virus isolation and qRT-PCR) in both the OPF **(A)** and Blood **(B)**. qRT-PCR is shown in pink and virus isolation method is shown in blue. The predicted line and 95% confidence intervals were done using PROC LOESS (SAS version 9.3). Smoothing parameter was 1.0 for OPF both VI and qRT-PCR and Blood qRT-PCR smoothing parameter for Blood VI was 0.67.

The pattern of virus detection with time (days) was similar for both the qRT-PCR and the virus isolation (VI) methods (Figure 3). Interestingly, virus in the OPF was always detected or not on a given day by both methods (Cohen’s kappa measure of agreement 1.00 (95% CIs 1.00-1.00)). However, agreement for detection of virus in the blood was lower (Cohen’s kappa 0.77 (95% CIs 0.62-0.93)). In general, in the blood low levels of virus were detected early post infection using the VI method when there was no virus detected using the qRT-PCR method. This may represent cell-associated virus. Viral genomes were detected using the qRT-PCR method late in study period, after there was no longer any virus detected using the VI method.

### Pre-clinical predictors

**Figure 4:**
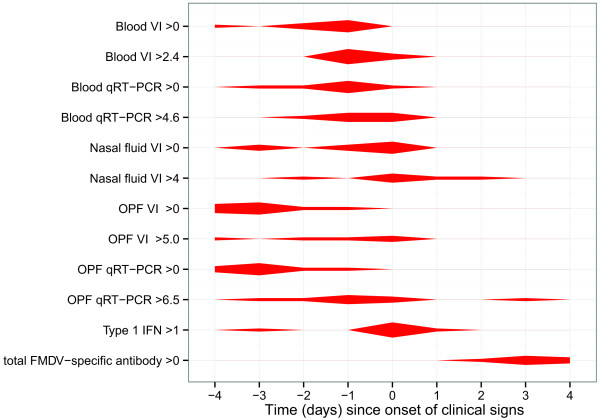
**Indicators of the onset of clinical signs.** A Violin plot of the appearance and onset of the immunological and virological variables tested in this study in relation to the onset of clinical signs.

Figure 4 is a violin plot representing the appearance of various predictors with reference to the time (days) since the onset of clinical signs. The thicker the line the more data within a given category are within the time frame specified. Very early in the infectious process OPF VI and OPF qRT-PCR were detectable whereas virus was not detected in the blood and nasal fluid until 2–3 days before the onset of clinical signs. Antibodies were not detected until 2–3 days after the onset of clinical signs.

In summary, virus is first detectable in the OPF, but detection of virus in the blood and nasal fluid may also be good candidates for preclinical indicators. Interestingly, the donor that did not transmit in this study never had any measurable amount of virus in the blood. However, virus was detectable in the OPF and nasal fluid.

### Temperature

**Figure 5:**
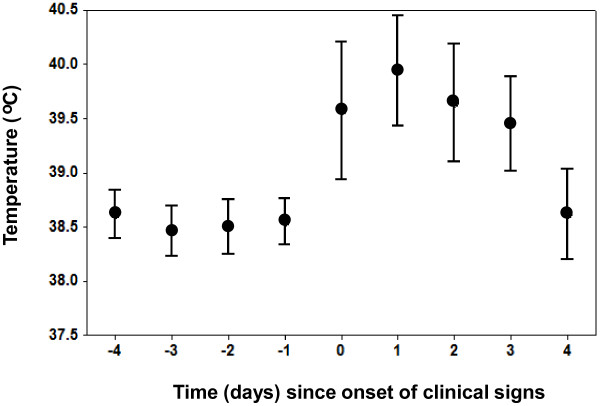
**Temperature.** Mean and 95% CIs for temperature (°C) in relation to the time (in days) since the onset of clinical signs.

A change in temperature was significantly associated with the onset of clinical signs (GLMM, *p* < 0.05). On the day that clinical signs appear temperature increases by an average of 1°C (Figure 5). Average temperature on the day that clinical signs appear was 39.6°C (95% CIs, 38.9°C-40.2°C) whereas the day before it was 38.6°C (95% CIs, 38.3°C-38.8°C).

## Discussion

Previous analysis of the experimental data used in this study showed that transmission was associated with onset of clinical signs [19]. In this study we further characterize this relationship and look for predictors of the onset of clinical signs as a proxy for transmission, thus increasing the statistical power to identify indicators of infectiousness. This is important because experiments with large animals held in high containment facilities are challenging and, inevitably, it is only feasible to use a low number of replicates. Despite this, we were able to identify factors significantly associated with different stages of FMDV infection. However, the association between transmission and the onset of clinical signs implies that relying on the detection of clinical infections will not facilitate the removal of infected animals before they become infectious, so preclinical diagnosis is required to achieve this.

All immunological and virological variables measured (with the exception of total FMDV-specific antibodies) were positively associated with onset of clinical signs. This result is not surprising given that they were chosen a priori as useful measures of the transmission of FMDV [19]. It does appear that onset of clinical signs only occur when virus levels exceed thresholds. This will surely be a useful measure for monitoring animals in further research programs and possibly in the field.

Air sample results were not included in previous analysis of this data [19] because problems associated with including missing data in an ordination analysis. This study, however, has shown that there is a significant association between virus detected in the air and transmission of FMDV. This does not mean that virus in the air is a vehicle of transmission in this study. We believe, however, that it is a marker for transmission. We note that the initial designs of this transmission study included an indirect transmission element (data not shown). Although airborne virus was detected in the air during the challenge periods no transmission occurred by this indirect route so further attempts at indirect transmission were not done. With such a small sample size the confidence limits are large, however, this possibly suggests that this is not a major route for disease spread between cattle, even though it appears to be an indicator of when an animal is infectious. Though the lack of airborne transmission might be due to the properties of this particular strain of virus as there was only a limited number of documented cases of airborne spread [40,41]. Planned future research will include more rigorous air sampling as these results suggest that air sampling shows great promise as a predictor of transmission and may also prove to be useful to detect preclinical infection. Hand held devices have been developed and their feasibility for monitoring shedding of FMDV in cattle is being tested [42].

Temperature was a good indicator of the onset of clinical signs and of transmission [19]. However, elevated temperatures do not occur early in the course of infection. Temperature is, of course, a non-specific clinical sign as such would have limited utility as a pre-clinical screening tool.

For OPF there was no difference between the two methods of virus detection. The levels recorded using qRT-PCR tended to be higher at the peak but for both methods there was perfect agreement with respect to detection of virus. Measurement of virus in the blood using the VI method resulted (in some cases) in low levels of virus being detected earlier. Virus was detectable by qRT-PCR (in some cases) even when it was not using the VI method, but this always occurred later in the course of infection. It is unknown whether the virus detected by qRT-PCR at these stages is inactivated or at such a low concentration that it is unable to be detected by virus isolation.

This analysis has confirmed the close association between the onset of clinical signs and the transmission of FMDV from an infected bovine reported previously [19]. In addition, we have identified predictors of clinical signs, namely virus present in the OPF, blood or nasal fluid but, importantly, only above a measured threshold. We also find that transmission is strongly associated with detectable levels of virus in the air although this need not imply that air-borne spread is itself a major route of transmission.

It has been argued that early detection of FMDV infection was critical to effective control of outbreaks and could help remove the need for pre-emptive culling [19]. As clinical signs appear very close to the onset of infectiousness these are not ideal indicators. Here, we report that the detection of virus in OPF provides the earliest indication; however, this is unlikely to be practicable in the field. Alternatively, detection of virus in the blood or nasal fluid is possible days before the appearance of clinical signs. In terms of early detection of infection, the VI method performs best but, in contrast to PCR methods, is not a good basis for a rapid penside test. In future work, given that we have demonstrated that virus can be detected early in the course of infection in OPF samples and nasal swabs, we intend to explore the possibility of developing more sensitive air sampling methods as the most obviously practicable approach to mass screening.

## Competing interests

The authors declare that they have no competing interests.

## Authors’ contributions

Conceived and designed the experiments: BC, MEJW, BMB, NDJ, PVB, SJC. Performed the experiments: BMB, NDJ, DG. Analyzed the data: MCT, IH. Contributed reagents/materials/analysis tools: BMB, NDJ, DG, MW, ER, SJC. Wrote the paper: MCT, MEJW. Revised the draft for important intellectual content: PB, BC, MEJW. Approved the final version for publication: BMB, NDJ, MCT, DG, SJC, MW, ER, PVB, RH, IH, MEJW, BC. All authors read and approved the final manuscript.

## Authors’ information

Paul V Barnett and Bryan Charleston are Jenner Institute investigators.
